# Supporting evidence-informed policy: a multidimensional impact analysis of health technology assessment in Thailand

**DOI:** 10.3389/fpubh.2026.1816280

**Published:** 2026-07-08

**Authors:** Thanisa Thathong, Khunjira Udomaksorn, Nusaraporn Kessomboon, Osot Nerapusee, Pattara Leelahavarong, Rungpetch C. Sakulbumrungsil

**Affiliations:** 1 Social and Administrative Pharmacy Department, Faculty of Pharmaceutical Sciences, Chulalongkorn University, Bangkok, Thailand; 2 Food and Drug Administration, Ministry of Public Health, Nonthaburi, Thailand; 3 Social and Administrative Pharmacy Department, Faculty of Pharmaceutical Sciences, Prince of Songkla University, Songkhla, Thailand; 4 Social and Administrative Pharmacy Department, Faculty of Pharmaceutical Sciences, Khon Kaen University, Khon Kaen, Thailand; 5 Social and Administrative Pharmacy Department, School of Pharmacy, Eastern Asia University, Pathum Thani, Thailand; 6 Siriraj Health Policy Unit, Faculty of Medicine Siriraj Hospital, Mahidol University, Bangkok, Thailand

**Keywords:** drug reimbursement, economic evaluation, health policy, health technology assessment, National List of Essential Medicines, Net Monetary Benefit, payback framework, Thailand

## Abstract

**Introduction:**

Health Technology Assessment (HTA) is widely used for evidence-informed priority setting in Thailand and has been integrated into pharmaceutical reimbursement decision-making through the National List of Essential Medicines (NLEM). However, its broader contributions have not been systematically assessed.

**Methods:**

We retrospectively analyzed 68 completed economic evaluations conducted between 2010 and 2018 using an adapted Payback Framework across five dimensions of impact: informing policy, knowledge production, capacity building, health sector benefit, and an economic dimension assessed separately for approved and rejected items using Net Monetary Benefit (NMB).

**Results:**

The principal finding concerns the economic dimension. Among 21 approved technologies, 12 (57.1%) had negative NMB at the national cost-effectiveness threshold, suggesting that adoption would not have been supported on cost-effectiveness grounds alone. Nevertheless, each of these approvals included a structured non-cost-effectiveness justification in committee deliberations, all of which mapped to recognized multi-criteria decision analysis domains, including severity, unmet clinical need, rarity, lack of alternatives, and standard-of-care positioning. This provides empirical evidence that deliberative multi-criteria reasoning operates in routine reimbursement practice in an institutionalized LMIC HTA-to-reimbursement pathway. Among 47 rejected items, 85.1% had positive modeled NMB under a non-adoption sign convention, indicating that non-adoption was consistent with efficient resource use at the threshold. The four categorical dimensions, on a 0-4 ordinal scale, characterized the cohort along complementary axes: informing policy (mean 3.34), health sector benefit (mean 2.54), capacity building (mean 1.97), and knowledge production (mean 1.87).

**Discussion:**

These findings suggest that HTA in Thailand contributes to disciplined, transparent multi-criteria priority setting. Aggregate NMB was concentrated in a few large-population, lifetime-horizon studies (14 accounting for 93.6% of the lifetime-horizon total), best interpreted as a descriptive summary rather than a uniform signal. The analytic approach may other insights for other low- and middle-income countries institutionalizing HTA within structured reimbursement systems.

## 1 Introduction

Global healthcare systems are currently grappling with an enduring fiscal paradox: the rapid acceleration of high-cost pharmaceutical innovation alongside the ethical and political imperative of achieving universal health equity (1). In response to escalating costs, nations have increasingly adopted structured, evidence-based frameworks to govern healthcare resource management (2). Health Technology Assessment (HTA) has emerged as a cornerstone of this transition, providing policymakers with a systematic, multidisciplinary process to balance clinical efficacy with economic sustainability (3, 4). By integrating medical, social, and economic perspectives, HTA supports rational priority setting and optimal resource allocation to maximize health gains within constrained budgets (3, 4). For low- or middle-income countries (LMICs), HTA is increasingly recognized as a key institutional mechanism for advancing and sustaining Universal Health Coverage (UHC) under conditions of finite fiscal space (5).

Thailand has been a regional pioneer in this domain, formally integrating HTA into its national health policy framework since 2007 (6). This integration was strategically designed to optimize healthcare resource allocation while ensuring equitable access to essential treatments across a diverse and aging population (6, 7). Thailand's healthcare delivery system is underpinned by three primary public insurance schemes: the Civil Servants Medical Benefit Scheme (CSMBS), covering government employees and their dependents; the Social Security Scheme (SSS) for formal private sector employees; and the Universal Coverage Scheme (UC), which covers the remainder of the population (8, 9). While these schemes serve different demographic segments, they are unified by the National List of Essential Medicines (NLEM), which functions as the national reimbursement list. HTA acts as an important component within the gatekeeping process of the NLEM, substantially influencing medicine accessibility and supporting fiscally responsible decision-making in the Thai health system (10).

Despite the acknowledged importance of HTA, comprehensive evaluations of its contributions within specific institutional reimbursement pathways remain scarce (11). Previous investigations have primarily focused on immediate policy outcomes or specific applications of HTA within the Thai context (12–14), without systematically examining its broader contributions to knowledge production, technical capacity building, and the economic dimension of reimbursement. This study addresses that gap by treating HTA as the primary evidence-generation mechanism of the NLEM reimbursement process and evaluating its multidimensional contributions accordingly. Using an adapted Payback Framework operationalized around NLEM institutional objectives rather than generic HTA impact concepts, we assessed the complete 9 year cohort of economic evaluations (EEs) commissioned within the pharmaceutical reimbursement pathway. This allows us to examine not only the timeliness and dissemination of HTA outputs, but also how the NLEM committee weighs cost-effectiveness evidence against other criteria when reaching reimbursement decisions, providing empirical insight into the practical operation of multi-criteria priority setting in an institutionalized LMIC HTA-to-reimbursement pathway. In particular, we foreground what we term the “Approved Paradox”: the observation that a majority of approved technologies did not meet the cost-effectiveness threshold on economic grounds alone, yet each such approval was accompanied by a structured, documented non-cost-effectiveness justification. This pattern is the finding that gives the economic analysis its interpretive weight and motivates the multidimensional approach adopted here.

## 2 Materials and methods

### 2.1 Theoretical framework: the payback framework

To evaluate the multidimensional contributions of EEs conducted within the Thai NLEM pharmaceutical reimbursement pathway, this study utilized an assessment tool adapted from the Payback Framework originally developed by Buxton and Hanney (15). The Payback Framework identifies five categories of research payback: knowledge; research benefits; political and administrative benefits; health sector benefits; and broader economic benefits. The framework conceptualizes research impact as progressing from initial inputs, defined as research needs, through the research process to these final outcomes.

All five original categories were retained in this study, with each operationalized for the institutional logic of the NLEM pharmaceutical reimbursement pathway rather than for generic HTA impact concepts. The five categories correspond to the manuscript‘s Knowledge Production, Capacity Building, Informing Policy Decision, Health Sector Benefit, and Economic Dimension respectively. Earlier applications of the framework typically used project-level case studies to trace pathways from research inputs and processes through to outputs, outcomes, and broader societal and economic impacts (15, 16). Subsequent applications have extended the framework to policy-oriented decision-making contexts: Klautzer et al. (17) demonstrated its use through categorical impact clusters, while Wooding et al. (18) applied it across a multinational cohort of cardiovascular and stroke research projects using systematic cross-case rating of all five payback categories. Following this pragmatic logic, our study operationalizes each category around the policy-facing decision objectives of the NLEM pathway. The detailed operationalization of each dimension is described in the Methods section.

### 2.2 Tool development

The assessment tool was operationalized by translating each Payback Framework category into a structured, criterion-referenced scoring system anchored in observable evidence drawn from documented committee decisions, dated meeting minutes, publication records, author and institutional records, and WHO ATC classification. The tool was developed through two preparatory steps prior to scoring.

First, a multi-stakeholder consultation meeting was convened with 30 participants drawn from public and private sector organizations involved in HTA in Thailand, including experts, researchers, and professional associations across multiple disciplines. The objective of the meeting was to endorse use of the Payback Framework as the theoretical foundation and to scope the assessment to the NLEM pharmaceutical reimbursement pathway.

Second, a focus group of seven HTA experts, all with established expertise in HTA and economic evaluation, was convened to operationalize each dimension and refine the rubric. Each dimension definition and each level of the 0–4 ordinal rubric was reviewed against pre-specified criteria of observability, reproducibility, and policy relevance. The output was a working version of the rubric, carried forward into the scoring application described in Section 2.6.

### 2.3 Study cohort

The assessment tool was retrospectively applied to a cohort of 68 EE cases conducted in Thailand between 2010 and 2018. These cases represented the complete official population of pharmaceutical products assigned by the NLEM committee or its coordinating working group for mandatory cost-effectiveness assessment during the study period. The cohort therefore captured EE studies conducted within a specific institutional pathway, the NLEM-mandated pharmaceutical cost-effectiveness assessment process, rather than the full population of HTA activities in Thailand. Within the cohort, some cases modeled the same eligible patient population across different clinical indications, reflecting the structure of pharmaceutical reimbursement requests in which a single drug class may be assessed for multiple indications within the same target population.

Data were extracted from the final comprehensive research reports submitted to the NLEM for reimbursement consideration, supplemented by documented committee decision records. Inclusion criteria were restricted to completed EE studies that had reached a definitive NLEM policy decision within the study period. This restriction reflects the outcome-based structure of the assessment tool, which requires finalized decision data to enable consistent scoring across all dimensions, particularly Dimension 1, Informing Policy Decision. Studies for which a definitive decision had not been reached were therefore excluded. Two studies were excluded on this basis. The potential effect of this exclusion on study findings is examined in the sensitivity analysis described in Section 2.7. The cohort represents the complete, closed population of NLEM-mandated pharmaceutical economic evaluations reaching a definitive committee decision within the 2010–2018 study period; because inclusion required a finalized decision, the dataset was fixed at the close of this period. The analysis was conducted in 2020–2021.

### 2.4 Categorical scoring rubric (dimensions 1–4)

The 68 evaluations were systematically scored based on specific criteria for four categorical dimensions. These dimensions were selected as pragmatic proxies for policy-relevant domains of impact in the Thai HTA context. Each dimension uses a five-point ordinal scale (0–4), where higher scores indicate greater observable impact within that domain.

Dimension 1: informing policy decision assesses the speed and directional alignment of EE evidence with the NLEM reimbursement decision. This dimension captures both timeliness and directional alignment as a composite indicator of policy responsiveness, reflecting the institutional reality that both components are relevant to the effectiveness of the evidence-to-policy pathway within the NLEM committee cycle.

- Score 4: decision consistent with study result; total time to decision not more than 3 years.
- Score 3: decision consistent with study result; total time to decision 4–6 years.
- Score 2: decision not consistent with study result; total time to decision not more than 3 years.
- Score 1: decision not consistent with study result; total time to decision 4–6 years.
- Score 0: total time to decision more than 6 years.

Dimension 2: knowledge production evaluates the dissemination and external reach of research outputs. This dimension measures dissemination mode as an observable proxy for external reach and knowledge translation rather than as a direct measure of scientific impact or methodological quality.

- Score 4: published in an international peer-reviewed journal.
- Score 3: published in a Thai peer-reviewed journal.
- Score 2: disseminated through conference proceeding or academic presentation.
- Score 1: internal technical report submitted to the NLEM.
- Score 0: no full report produced.

Dimension 3: capacity Building measures organizational collaboration and human resource development. This dimension captures collaboration structure and human resource involvement as observable proxies for sustainable HTA capacity development. The NLEM evaluation process serves as the institutional vehicle, but the capacity built through multi-organizational engagement and involvement of students and practitioners contributes to the broader national HTA ecosystem beyond the NLEM process itself.

- Score 4: conducted with participation of more than one organization, with both students and practitioners involved.
- Score 3: conducted with participation of more than one organization, with at least one student or practitioner involved.
- Score 2: conducted with participation of more than one organization, with no students or practitioners involved.
- Score 1: conducted within a single organization, with at least one student or practitioner involved.
- Score 0: conducted within a single organization, with no students or practitioners involved.

Dimension 4: health sector benefit captures therapeutic novelty of the evaluated medicine for its studied clinical condition, classified using the Anatomical Therapeutic Chemical (ATC) system. This dimension operationalizes therapeutic novelty using ATC classification as a structured proxy for the relative positioning of medicines within existing treatment options, rather than as a direct measure of clinical benefit or innovation.

- Score 4: First medicine evaluated for the studied indication.
- Score 3: First medicine of the chemical subgroup (ATC 4th level) for the studied indication.
- Score 2: Second medicine evaluated for the studied indication.
- Score 1: Second medicine of the chemical subgroup (ATC 4th level) for the studied indication.
- Score 0: later medicine of the chemical subgroup or evaluation for an existing indication.

### 2.5 Dimension 5: economic dimension (Net Monetary Benefit)

The Economic Dimension was operationalized using the Net Monetary Benefit (NMB) framework, the linear net-benefit formulation introduced by Stinnett and Mullahy (19) as an alternative to ratio-based metrics in cost-effectiveness analysis. Because approved and rejected technologies represent fundamentally different analytic contexts, NMB was calculated and reported separately for each group. The approved-item analysis is grounded in observed reimbursement decisions and submitted model parameters, while the rejected-item analysis is counterfactual, requiring assumptions about uptake that cannot be empirically observed. The two sets of estimates reflect differing interpretive questions and are not intended to be pooled or directly compared as a single aggregate.

For approved items, NMB was calculated to assess whether the health gains associated with each approved technology justified its incremental cost under the national cost-effectiveness threshold:


$$\begin{array}{l} \text{NMB }=\;\left(\text{Δ}QALYs\;\times \;P\;\times \;CET\right)\;-\left(\Delta Costs\;\times P\right) \end{array}$$

A positive NMB indicates that the modeled health gains outweigh the incremental costs at the threshold, supporting the cost-effectiveness of the reimbursement decision.

For rejected items, NMB was calculated using the same model inputs under a non-adoption sign convention, such that a positive value indicates that non-adoption was directionally consistent with efficient resource use at the national threshold:


$$\begin{array}{l} \text{NMB }=\;\left(\text{Δ}Costs\;\times \;P\right)\;-\;\left(\Delta QALYs\;\times \;P\;\times \;CET\right) \end{array}$$

This presentation preserves sign-directionality across both groups, so that a positive NMB can be read consistently as supporting the observed decision, adoption in the approved group, non-adoption in the rejected group.

In both formulas, ΔQALYs denotes the incremental quality-adjusted life years, ΔCosts denotes the incremental cost, P denotes the estimated number of target patients over the specified analytic time horizon as reported in the original submitted model, and CET denotes the official Thai cost-effectiveness threshold of 160,000 THB per QALY (approximately USD 5,097 per QALY).

For each included case, the NMB calculation used the incremental costs, incremental QALYs, target patient population, and analytic time horizon as reported in the final submitted EE report. These inputs were extracted directly from the original evaluations without modification. All included EEs were conducted in accordance with the Thai HTA process guideline (12) and applied the recommended discount rate specified in that guideline. Because the included studies differed in model structure, target population, disease context, and analytic time horizon, the NMB estimates were not re-standardized to a common analytic framework and are reported using the parameters of each original submitted evaluation.

For rejected items, the base case NMB was calculated under the 100% target population coverage assumption embedded in the original submitted models, consistent with standard practice in economic evaluation models submitted for reimbursement appraisal. The effect of lower uptake assumptions on rejected-item NMB is examined in the sensitivity analysis described in Section 2.7.

All analyses were conducted in Thai baht. For presentation, NMB values were additionally expressed in million USD using a single fixed exchange rate of 31.39 THB per USD, corresponding to the time of manuscript preparation in 2021 and consistent with the official conversion of the national cost-effectiveness threshold (160,000 THB ≈ USD 5,097 per QALY). Because this conversion is a one-time convenience rescaling of the underlying THB estimates rather than a present-value comparison across time periods, a single fixed rate was applied uniformly across all cases and no inflation adjustment was made. This rate remains broadly representative of the THB/USD exchange rate over the study and contemporary periods, indicating that the USD-denominated estimates are not materially sensitive to the choice of reference year.

### 2.6 Scoring application and validation

The working rubric developed in Section 2.2 was applied to the cohort of 68 EE cases through a three-tier review process across five reviewers. A primary researcher assigned scores from full case documentation against the rubric. A second researcher reviewed each score for consistency with the rubric and flagged cases where the rubric itself appeared discordant or inappropriate. A panel of three HTA experts provided final confirmation through collective deliberation. During an initial pilot phase, scoring revealed levels that required clarification or scale calibration; the rubric was refined by panel consensus and previously scored cases were re-reviewed under the refined rubric. This iterative process continued until the rubric was stable across the full cohort, after which the final scoring round produced the dataset reported in the Results. This multi-tier iterative scoring process is consistent with previous applications of the Payback Framework that have used expert panels with two-round rating and structured discussion to enhance consistency in cross-case impact assessment (18). Because the four categorical dimensions are scored against objective, externally documented attributes, decision dates relative to fixed time thresholds, dissemination venue, the documented composition of the research team, and ATC classification, score assignment is largely deterministic given the source documentation and leaves limited scope for subjective rater interpretation. The discordances that arose during the pilot phase were therefore pre-dominantly definitional, concerning where a category boundary should be set, rather than disagreements over the application of a fixed rubric, and were resolved by refining the criteria themselves. For this reason, a formal inter-rater agreement statistic was not computed and an itemized log of individual disagreements was not maintained; the process was one of iterative rubric specification followed by consensus confirmation rather than independent parallel rating. The reproducibility of the finalized rubric in the hands of fully independent raters is addressed in the Limitations.

After scoring was complete, a stakeholder hearing was convened with 32 participants, including senior policy makers, members of the NLEM working groups, and representatives of all three major Thai public health insurance schemes (CSMBS, SSS, and UC). This hearing served as a face-validity and policy-relevance check on the interpretation of the findings, distinct from the expert-panel verification of the scoring described above; the stakeholders endorsed the findings without revision and provided comments on their interpretation and policy implications.

### 2.7 Sensitivity analyses

Two pre-specified sensitivity analyses were conducted to examine the robustness of primary findings to key analytic boundary assumptions.

#### 2.7.1 Sensitivity analysis 1: effect of excluded cases on dimension 1 scoring

Two EE studies were excluded from the primary cohort because a definitive NLEM decision had not been reached within the study period. To examine the potential effect of this exclusion, both excluded cases were assigned the minimum possible score of 0 for Dimension 1 and the dimension mean was recalculated, providing the most conservative plausible estimate, as it assumes complete absence of timely policy influence for the excluded cases. This analysis was restricted to Dimension 1, as the scoring criteria for Dimensions 2–4 are independent of NLEM decision status. For Dimension 5, a corresponding sensitivity analysis was not feasible, as NMB calculation requires knowledge of the observed decision direction (approval or rejection), which cannot be inferred without introducing additional untestable assumptions.

#### 2.7.2 Sensitivity analysis 2: effect of alternative uptake assumptions on rejected-item NMB

Three alternative uptake scenarios, 25, 50, and 75% of the original target patient population specified in each submitted model, were applied to rejected cases to span a plausible range of counterfactual utilization patterns in real-world settings. This subgroup-adoption sensitivity structure is consistent with the framework developed by Kim and Basu (20), which formalizes aggregate population-level net benefit as a function of subgroup-specific adoption parameters and argues that policy-relevant economic evaluation under heterogeneity requires explicit modeling of uptake rather than reliance on the implicit 100% adoption assumption embedded in submitted models. The scenarios are treated as equally weighted; no single scenario is designated as a reference or best estimate. The base case 100% figure, derived from the full target population coverage assumption of the original submitted models, is retained as the upper bound of the sensitivity range. Approved cases retained the parameters of the underlying submitted economic evaluations across all scenarios.

## 3 Results

The retrospective multidimensional assessment of 68 EE cases provides empirical insights into HTA's contribution to pharmaceutical reimbursement decision-making in Thailand across five dimensions of impact. The following sections present findings for the four categorical dimensions and the Economic Dimension separately.

### 3.1 Multidimensional impact scores (dimensions 1–4)

**Figure 1:**
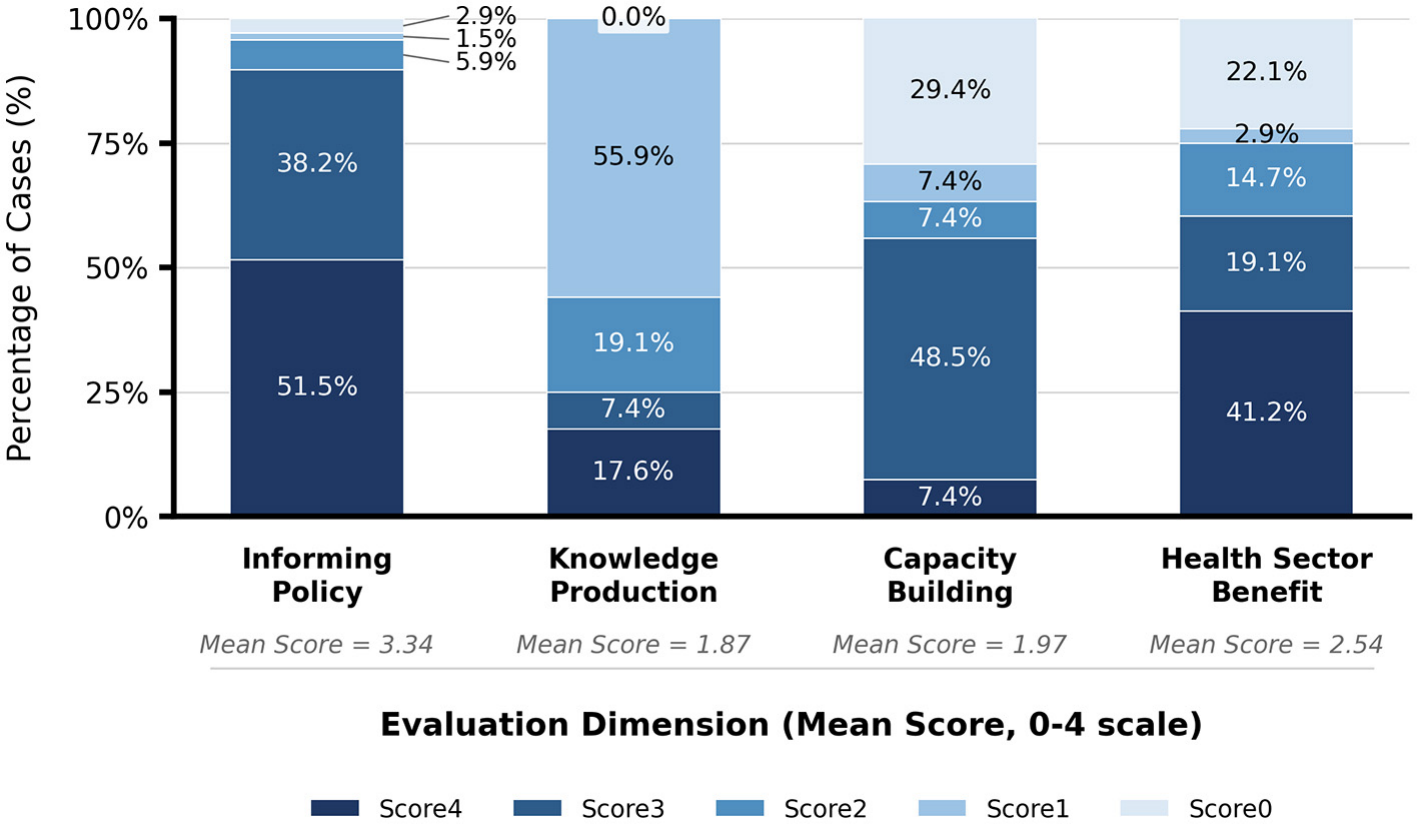
Distribution of categorical impact scores across four dimensions of the adapted Payback Framework (*N* = 68). Score 4 indicates maximal impact; Score 0 indicates minimal impact. Mean scores are shown below each dimension label.

The distribution of scores across all four categorical dimensions is presented in Table 1 and Figure 1.

**Table 1:** Distribution of categorical impact scores across four dimensions (*N* = 68).

Dimension	Mean score	Score 4 (maximal)	Score 3	Score 2	Score 1	Score 0 (minimal)
Informing policy	3.34	51.5% (*n* = 35)	38.2% (*n* = 26)	5.9% (*n* = 4)	1.5% (*n* = 1)	2.9% (*n* = 2)
Knowledge production	1.87	17.6% (*n* = 12)	7.4% (*n* = 5)	19.1% (*n* = 13)	55.9% (*n* = 38)	0.0% (*n* = 0)
Capacity building	1.97	7.4% (*n* = 5)	48.5% (*n* = 33)	7.4% (*n* = 5)	7.4% (*n* = 5)	29.4% (*n* = 20)
Health sector benefit	2.54	41.2% (*n* = 28)	19.1% (*n* = 13)	14.7% (*n* = 10)	2.9% (*n* = 2)	22.1% (*n* = 15)

Dimension 1 (informing policy decision) was the highest-performing dimension, with a mean score of 3.34. Over half of the studies (51.5%, *n* = 35) achieved the maximum score of 4, indicating that evidence was associated with policy decisions within a 3-year NLEM committee cycle. Only 2.9% of cases (*n* = 2) required more than 6 years to reach a decision. In the sensitivity analysis assigning both excluded cases a minimum score of 0, the mean score decreased modestly from 3.34 to 3.24.

Dimension 2 (knowledge production) was the lowest-performing dimension, with a mean score of 1.87. A majority of HTA outputs (55.9%, *n* = 38) remained as internal technical reports submitted to the NLEM, while 17.6% (*n* = 12) were published in international peer-reviewed journals. This pattern reflects limited external dissemination rather than an absence of formal methodological review, as all reports were developed and appraised in accordance with national HTA guidelines before committee deliberation.

Dimension 3 (capacity building) showed the second-lowest performance, with a mean score of 1.97. Although 48.5% (*n* = 33) of studies involved multi-organizational collaboration with at least one student or practitioner, only 7.4% (*n* = 5) achieved the highest score by involving both students and practitioners across multiple organizations. At the other end of the distribution, 29.4% (*n* = 20) were conducted by a single organization without student or practitioner involvement.

Dimension 4 (health sector benefit) had a mean score of 2.54. A total of 41.2% (*n* = 28) of evaluations involved first-in-class therapies for their studied indication, and collectively, over 60% of evaluations involved medicines representing newer therapeutic options within the ATC classification. The full score distributions and mean scores across all four dimensions are presented in Figure 1.

### 3.2 Economic dimension (dimension 5): NMB of approved technologies and NMB under hypothetical uptake scenarios of rejected technologies

#### 3.2.1 NMB of approved technologies

Among the 21 approved items, 12 (57.1%) yielded negative NMB at the national cost-effectiveness threshold of 160,000 THB (USD 5,097) per QALY, indicating that the submitted cost-effectiveness evidence alone would not have supported their adoption. The remaining nine approvals (42.9%) yielded positive NMB consistent with cost-effectiveness at the threshold. The aggregate approved-item NMB across all 21 approvals was USD 153.10 million (Table 2).

**Table 2:** Net Monetary Benefit (NMB) by NLEM decision status.

NLEM decision	Cases *N* = 68	Positive NMB *N* = 49 (*n*, %)	Negative NMB *N* = 19 (*n*, %)	Approved-item aggregate NMB (mUSD)	Rejected-item modeled aggregate NMB (mUSD)
Approved	21	9 (42.9%)	12 (57.1%)	$153.10	Not applicable
Rejected	47	40 (85.1%)	7 (14.9%)	Not applicable	$5,686.56

Approved-item and rejected-item NMB analyses answer different analytic questions and are reported in separate columns.

The approved-item NMB is grounded in observed reimbursement decisions; the rejected-item NMB is a counterfactual scenario calculation applying the inverted-sign formula (Section 2.5) to submitted model parameters under a 100% target population coverage assumption.

Sensitivity analysis under alternative uptake assumptions is presented in Table 4.

The occurrence of negative NMB was not uniformly distributed across therapeutic areas: the highest proportions were observed in rare diseases (100%, 3/3), respiratory disease (100%, 1/1), and oncology (62.5%, 5/8); moderate proportions in infectious diseases and neurology (50% each); a lower proportion in autoimmune and immunologic diseases (33%, 1/3); and no negative-NMB approvals in the endocrine/metabolic or non-cancer hematology categories. Each of the 12 negative-NMB approvals was accompanied by a structured non-cost-effectiveness justification documented in NLEM committee records, and every justification mapped onto recognized multi-criteria decision analysis criteria including severity, unmet clinical need, rarity, absence of therapeutic alternatives, and standard-of-care positioning (Table 3).

**Table 3:** Approved items with negative Net Monetary Benefit (NMB) by therapeutic area, with multi-criteria decision analysis (MCDA) mapping.

Therapeutic area	Total approved (*n*)	Negative NMB (*n*)	Negative NMB (%)	Documented NLEM committee rationale	MCDA criteria identified
Oncology	8	5	62.5	Included therapies recognized as standard of care in specific clinical settings, including first-line regimens such as rituximab for diffuse large B-cell lymphoma (DLBCL)	Standard-of-care status; severity of disease
Rare diseases	3	3	100	Included therapies for conditions with very small patient populations and limited or no alternative treatment options, such as imiglucerase for Gaucher disease	Rarity; absence of therapeutic alternatives; unmet clinical need
Autoimmune/Immunologic diseases	3	1	33.3	Included therapies used in patients with inadequate response to first-line treatment	Second-line/refractory positioning; unmet clinical need
Infectious diseases	2	1	50	Included therapies considered necessary for disease control in specific patient groups	Disease-control necessity; unmet clinical need
Neurology	2	1	50	Included therapies providing symptomatic benefit in chronic progressive conditions	Severity of disease; symptomatic benefit in progressive conditions
Respiratory disease	1	1	100	Included therapies used in severe disease with substantial unmet clinical need	Severity of disease; unmet clinical need
Endocrine/metabolic disease	1	0	0	No approved item with negative NMB in this category	Not applicable
Hematology (non-cancer)	1	0	0	No approved item with negative NMB in this category	Not applicable

The fifth column summarizes the documented rationale for approval as recorded in NLEM committee decision documents.

The sixth column maps each rationale against pre-specified MCDA criteria drawn from the priority-setting literature (21, 22): severity and unmet clinical need, absence of therapeutic alternatives, rarity of the eligible population, standard-of-care status, and second-line or refractory clinical positioning.

All negative-NMB approvals were associated with at least one of these criteria, based on documented committee records.

MCDA criteria: Severity / unmet clinical need = high disease burden or absence of adequate symptom or disease control.

Absence of therapeutic alternatives = no reimbursed treatment option for the indication.

Rarity = very small eligible patient population.

Standard-of-care status = therapy recognized as the established standard for the clinical setting.

Second-line / refractory positioning = therapy used after inadequate response to first-line treatment.

#### 3.2.2 Modeled NMB of rejected technologies under hypothetical uptake scenarios

For the 47 rejected items, actual post-rejection uptake could not be observed. A scenario-based NMB model was therefore constructed to estimate the potential economic implications of non-adoption under a range of hypothetical uptake assumptions, representing projected counterfactual impact under modeled conditions.

Among rejected cases, 85.1% (*n* = 40) yielded a positive modeled NMB, suggesting that these technologies were unlikely to be cost-effective at the national threshold and that non-adoption was directionally consistent with efficient resource use. The three equally weighted uptake scenarios of 25, 50, and 75% yielded aggregate modeled NMB estimates of USD 1,421.64, USD 2,843.28, and USD 4,264.92 million respectively (Table 4). The directional consistency of positive modeled NMB across all three scenarios indicates that the finding is not driven by any single uptake assumptions, even though the magnitude varies substantially across the range. The base case 100% uptake scenario, derived from the full target population coverage assumption of the original submitted models, is retained in Table 4 as the upper bound of the sensitivity range.

**Table 4:** Modeled Net Monetary Benefit (NMB) of rejected technologies under alternative uptake assumptions.

Scenario	NMB of rejected items (mUSD)
25% uptake	1,421.64
50% uptake	2,843.28
75% uptake	4,264.92
100% uptake (base case from original model)	5,686.56

The three uptake scenarios are treated as equally weighted; no single scenario is designated as a reference or best estimate.

The 100% base case is derived from the full target population coverage assumption of the original submitted models and represents the upper bound of the sensitivity range, not a plausible estimate of real-world utilization.

#### 3.2.3 Heterogeneity in NMB estimates

Table 5 summarizes the analytic time horizons, patient population ranges and medians, and horizon-stratified NMB values across all included cases. Most studies (54/68) applied a lifetime horizon, while the remaining cases applied fixed time horizons ranging from 3 to 50 years. Studies with shorter time horizons (3–20 years) were pre-dominantly in oncology and hepatorenal syndrome. Within the 3, 5, and 50-year horizon categories, multiple studies modeled the same eligible patient population across different clinical indications, accounting for the identical population figures shown in Table 5. The majority of the aggregated NMB (combining approved-item NMB and rejected-item modeled NMB) was derived from lifetime-horizon studies across multiple therapeutic areas.

**Table 5:** Distribution of included cases by analytic time horizon, patient population, and total NMB.

Time horizon	Number of cases (*N* = 68)	Patient population range	Median of patient population	Therapeutic area	NMB (mUSD)
3 year	2	6,256–6,256	6,256	Cancer	38.92
5 year	3	4,122–4,122	4,122	Cancer	101.07
10 year	2	80–258	168	Cancer	12.51
15 year	2	80–1,840	960	Cancer	−23.28
20 year	1	110	110	Hepatorenal syndrome	0.32
50 year	4	28,563–33,003	28,563	Diabetes	381.35
Lifetime	54	2–266,300	1,008	Multiple therapeutic areas	5,328.79
Subgroup within lifetime cases	9	< 100 patients	42		−9.76
Subgroup within lifetime cases	31	100–10,000 patients	958		351.35
Subgroup within lifetime cases	14	>10,000 patients	18,536		4,987.20

Studies within the 3, 5, and 50-year horizon categories share target patient populations because multiple studies modeled different clinical indications within the same eligible population.

The final three rows present a subgroup analysis of the 54 studies using a lifetime horizon, categorized by target patient population size.

Median population sizes are reported alongside population ranges to characterize the distribution within each subgroup.

Within the lifetime-horizon subgroup, studies involving more than 10,000 patients (*n* = 14) contributed USD 4,987.20 million to the total NMB, 93.6% of the lifetime-horizon aggregate, while studies involving 100 to 10,000 patients (*n* = 31) contributed USD 351.35 million, and studies involving fewer than 100 patients (*n* = 9) contributed -USD 9.76 million. This pattern indicates that the aggregate NMB was driven primarily by studies with large eligible populations rather than reflecting a uniform pattern across cases.

## 4 Discussion

This study evaluated the contributions of EE studies within the Thai NLEM pharmaceutical reimbursement pathway using an adapted Payback Framework (15), with each dimension grounded in observable outcomes drawn from documented evidence and decisions. This approach is consistent with policy-oriented applications of the framework, including the work of Klautzer et al. (17), which emphasized examining research contributions through practically relevant domains in decision-making settings. The discussion proceeds as follows. Section 4.1 considers the policy responsiveness and dissemination findings; Section 4.2 considers the economic dimension and the heterogeneity of NMB estimates; Section 4.3 develops the central empirical contribution of the study, the structured pattern of non-cost-effectiveness justifications accompanying every threshold-departing approval; Section 4.4 considers implications for HTA development; Section 4.5 identifies areas for improvement; and Section 4.6 sets out the study‘s limitations.

### 4.1 HTA and policy responsiveness in Thailand

The Informing Policy dimension achieved the highest performance across all dimensions, indicating a high degree of alignment between economic evidence and final decisions. More than half of the analyzed studies (51.5%) were aligned with policy decisions within a 3-year timeframe, corresponding to the administrative term of the NLEM committee. This finding suggests that HTA is well integrated into domestic decision-making processes. At the same time, continued efforts to streamline timelines may remain important, particularly where delays could affect timely patient access.

The relatively low score in the Knowledge Production dimension primarily reflected the mode of dissemination rather than an assessment of methodological quality. Within the Thai NLEM process, EE studies are conducted in accordance with national HTA methodological guidelines and undergo structured methodological review by internal and external reviewers before committee deliberation (12). The pre-dominance of internal reports therefore indicates limited external dissemination and international visibility rather than insufficient technical rigor. This pattern suggests an opportunity to strengthen publication and knowledge translation pathways so that domestic HTA outputs contribute more visibly to the broader international literature.

### 4.2 Economic dimension (net monetary benefit, NMB)

The Economic Dimension of HTA contributions was assessed through two conceptually distinct analyses reported separately. For approved technologies, the NMB analysis draws directly on submitted cost-effectiveness evidence and provides an account of the economic implications of reimbursement decisions under the national threshold. For rejected technologies, the NMB analysis is counterfactual: it models the economic implications of non-adoption under hypothetical uptake scenarios, given that actual post-rejection utilization cannot be observed. The former is grounded in observed decisions and submitted data, the latter in scenario modeling under unobservable uptake assumptions, and the two sets of estimates are reported as separate analyses rather than as a single aggregate.

The findings suggest that one potential economic contribution of EE studies within the NLEM pathway lies in identifying technologies that are not cost-effective at the national threshold. The directional consistency of this finding across all three equally weighted uptake scenarios indicates robustness to uptake assumptions, although the magnitude of modeled estimates varies substantially across the range and represents projected counterfactual economic implications under hypothetical uptake conditions rather than realized savings.

The horizon-stratified and population-subgroup analyses indicate that the aggregated approved-item and rejected-item modeled NMB estimates were shaped by substantial structural differences across included studies. Within the lifetime-horizon subgroup, which accounted for most of the cohort, the 14 studies involving more than 10,000 patients (median population 18,536) contributed 93.6% of the lifetime-horizon aggregate NMB, while the 31 studies with 100–10,000 patients (median 958) and the nine studies with fewer than 100 patients (median 42) contributed proportionally less. Studies with shorter time horizons and smaller populations similarly contributed only marginally to the aggregate figure. An additional layer of structural variation is reflected in studies within the shorter fixed-horizon categories, where multiple cases modeled the same eligible patient population across different clinical indications. This pattern is consistent with the methodological argument of Kim and Basu (20) that population-level net benefit cannot be interpreted as a uniform signal in the presence of heterogeneity in subgroup composition, model structure, or adoption. Their framework formalizes aggregate NMB as a weighted function of subgroup-specific net benefits and adoption parameters; in the present cohort, the analogous sources of structural variation, including analytic time horizon, eligible population size, disease context, and indication breadth, each contribute differentially to the aggregate figure, confirming that these aggregates represent descriptive summaries of heterogeneous modeled estimates across structurally diverse technologies rather than a uniform signal across cases.

### 4.3 The “approved paradox” and multi-criteria reimbursement in practice

A key implication of this study is that EE is influential, but not determinative, in reimbursement decision-making. Although HTA appears effective in identifying economically favorable technologies, 57.1% of approved items yielded a negative NMB at the national threshold, meaning that the submitted cost-effectiveness evidence alone would not have supported adoption. This “Approved Paradox” is analytically meaningful not because deviations from the threshold occurred, but because they occurred in systematically identifiable clinical and policy contexts.

The subgroup analysis mapped the documented NLEM committee rationales for the 12 negative-NMB approvals (Table 3) against a pre-specified set of non-cost-effectiveness-analysis (non-CEA) criteria commonly applied in the multi-criteria decision analysis literature (21, 22): severity and unmet clinical need, absence of therapeutic alternatives, rarity of the eligible population, standard-of-care status, and second-line or refractory clinical positioning. Every negative-NMB approval was associated with at least one of these criteria, and most with two or more. Approvals in rare diseases (3/3) reflected rarity of the eligible population, absence of therapeutic alternatives, and unmet clinical need. Oncology approvals (5/8) reflected standard-of-care status combined with severity of disease, as in first-line regimens such as rituximab for diffuse large B-cell lymphoma. The respiratory disease approval reflected severity of disease and substantial unmet clinical need. Autoimmune and immunologic approvals reflected second-line or refractory positioning combined with unmet clinical need. Neurology approvals reflected severity of disease and symptomatic benefit in chronic progressive conditions. Infectious disease approvals reflected disease-control necessity in defined clinical groups together with unmet clinical need. No negative-NMB approval was documented without a structured non-CEA justification.

This structured pattern distinguishes the Approved Paradox from simple threshold inconsistency. In a reimbursement system that relied on cost-effectiveness alone, the 12 approvals would appear inconsistent with the national threshold and could be interpreted as decision errors. In a reimbursement system that applies deliberative multi-criteria reasoning, they represent the coherent operation of the system under circumstances in which cost-effectiveness is one input among several. The pattern observed in Thailand is consistent with the latter.

This pattern is also consistent with the wider priority-setting literature. A systematic review of LMIC priority-setting criteria reported that, in addition to cost-effectiveness and health benefits, factors such as equity, severity of disease, and implementation considerations are frequently described in the literature as criteria considered in priority-setting decisions (21). Work on reimbursement pathways for high-cost medicines has shown that technologies not meeting conventional value-for-money thresholds may still be covered under specific conditions, including life-saving benefit, absence of therapeutic alternatives, and affordability constraints (23). The distinctive contribution of the present study is empirical: it documents the criteria that were actually applied in a mature institutionalized HTA-to-reimbursement pathway over a 9-year period, rather than describing the criteria that priority-setting systems intend to use. Every deviation from the cost-effectiveness threshold was associated with a structured clinical or policy justification recorded in committee deliberations.

The implication for HTA system design is that the value of cost-effectiveness evidence is not undermined when the threshold is not met. Rather, transparent documentation of why the threshold was departed from, as observed in the Thai process, is what allows multi-criteria priority setting to remain disciplined, auditable, and publicly defensible. For other LMICs institutionalizing HTA, this suggests that building capacity for explicit recording of non-CEA rationales may be as important as building analytic capacity for the cost-effectiveness evidence itself.

### 4.4 Implications for HTA development and broader applicability

Taken together, these findings suggest that an institutionalized HTA system linked to a structured reimbursement process can support priority setting by incorporating economic evidence alongside broader considerations such as equity, severity of disease, unmet need, and implementation feasibility. Strengthening HTA systems requires attention not only to analytic capacity, but also to transparency, collaboration, and the communication of evidence beyond domestic decision processes.

While Thailand has an institutionalized HTA system linked with its reimbursement processes, the governance structures and decision criteria underpinning this system may not be directly replicable in all LMIC settings. The multidimensional framework used in this study is nonetheless adaptable to other contexts as a way of examining HTA contributions beyond individual coverage decisions. The direction of the policy lessons, the importance of linking HTA to structured priority setting, transparency, and deliberative decision-making, is more transferable than the magnitude of the economic estimates reported here. The analytic approach may therefore be informative for other LMICs seeking to institutionalize HTA within structured reimbursement frameworks. The institutional architecture linking HTA to NLEM reimbursement, mandatory economic evaluation of candidate high-cost medicines, deliberation by the NLEM subcommittee and its coordinating working groups, and reference to a published national cost-effectiveness threshold, has remained stable at 160,000 THB/QALY in its essential features over the period since the cohort closed (24). The institutional logic characterized in this study therefore remains representative of the operative reimbursement pathway. In short, the transferable elements lie in the policy logic and analytic approach, linking economic evidence to deliberative multi-criteria priority setting through transparent documentation, rather than in the Thai institutional architecture itself.

### 4.5 Areas for improvement and future directions

Despite these achievements, several gaps remain. First, many HTA outputs remain as internal technical reports rather than being disseminated through peer-reviewed publications, limiting their international visibility and contribution to the global HTA evidence base. Second, Capacity Building showed a concentrated distribution of performance, with only 7.4% of evaluations achieving the strongest structure, multi-organizational collaboration with both students and practitioners involved, and 29.4% conducted within a single organization without student or practitioner involvement. This distribution suggests that while multi-organizational collaboration is common, the combination of cross-institutional engagement and structured trainee involvement remains limited, indicating a need for broader multi-institutional collaboration and a more sustainable pipeline of trained expertise. This pattern likely reflects structural features of the NLEM process: economic evaluations are centrally commissioned within time-bound submission cycles, and HTA expertise in Thailand remains concentrated in a small number of institutional centers, which can favor lead institutions with established analytic capacity over distributed multi-institutional teams that include trainee participation. Strengthening this dimension may therefore require adjustments at the commissioning stage, such as longer lead times and explicit incentives for cross-institutional and trainee involvement, alongside broader investment in HTA training pipelines. Finally, as reimbursement decisions increasingly involve social and ethical trade-offs, more systematic documentation of stakeholder engagement, research prioritization, and committee reasoning would strengthen transparency and public trust in the NLEM process.

### 4.6 Study limitations

This study has several limitations.

First, its retrospective design relied on documented outcomes and may not have fully captured dynamic, real-time factors influencing policy decisions. Informal deliberations and variations in documentation quality across cases may not be fully reflected in the quantitative impact scores.

Second, restricting the cohort to cases with finalized policy decisions may have introduced upward bias in Dimension 1 scores. In the sensitivity analysis assigning the two excluded cases the minimum score of 0, the Dimension 1 mean decreased from 3.34 to 3.24, suggesting a modest numerical effect; however, broader upward bias cannot be excluded if unresolved cases differed systematically from included cases in policy traction or documentation quality. For Dimension 5, a corresponding sensitivity analysis was not feasible, as NMB calculation requires the decision direction, which is the specific information unavailable for excluded cases.

Third, actual post-rejection utilization could not be observed and was therefore modeled using hypothetical uptake scenarios, yielding projected counterfactual estimates rather than observed economic outcomes. The three equally weighted scenarios span a plausible range but cannot be validated against real-world utilization data. Heterogeneity in model structure, target population, disease context, and analytic time horizon further limits the strict comparability of aggregated NMB estimates: as shown in Table 5, lifetime-horizon studies involving more than 10,000 patients (median population 18,536) contributed 93.6% of the lifetime-horizon aggregate, and several shorter-horizon studies modeled the same eligible population across different clinical indications. Aggregate NMB figures should therefore be read as descriptive summaries of heterogeneous modeled estimates rather than uniform signals across the cohort. This interpretive caution aligns with the broader methodological literature on net-benefit aggregation under heterogeneity (19, 20), in which aggregate net benefit is treated as a structural summary of subgroup composition rather than a population-uniform parameter.

Fourth, the scoring rubric was developed as a pragmatic, policy-oriented assessment tool to support transparent multidimensional impact assessment within the NLEM pharmaceutical reimbursement pathway, rather than as a formally validated psychometric instrument. Although the rubric was anchored in documentary and internationally coded evidence and applied through three-tier verification with iterative pilot refinement (Section 2.6), the reproducibility of the final rubric in the hands of fully independent raters who were not part of the development process has not been empirically tested. Future applications of this or similar rubrics would benefit from building parallel independent scoring and quantitative reliability reporting into the protocol from the outset.

## 5 Conclusion

This study assessed the multidimensional contributions of economic evaluation to pharmaceutical reimbursement decision-making in Thailand using an adapted Payback Framework. The findings indicate that HTA functions as a disciplined and transparent input into NLEM decision-making, with cost-effectiveness evidence weighed alongside clinical and equity criteria in a structured rather than *ad hoc* manner.

Methodologically, this study demonstrates the value of combining two established but typically separate approaches—the Payback Framework for multidimensional impact assessment and Net Monetary Benefit analysis for estimating economic implications—within a single evaluation of an institutionalized HTA-to-reimbursement pathway.

Beyond this methodological contribution, three substantive implications follow. For the priority-setting literature, the documented pattern of structured non-cost-effectiveness justifications accompanying every threshold-departing approval provides empirical confirmation that multi-criteria reasoning is not merely an aspirational framework but a documented feature of mature LMIC reimbursement practice. For HTA system design, the disciplined documentation of why the cost-effectiveness threshold was departed from is what allows multi-criteria priority setting to remain auditable and publicly defensible; for other LMICs institutionalizing HTA, building capacity for explicit recording of non-CEA rationales may be as important as building analytic capacity for the cost-effectiveness evidence itself.

To strengthen the long-term contribution of HTA in Thailand specifically, future efforts should focus on expanding international dissemination of domestic outputs and supporting multi-institutional capacity building, while preserving the deliberative structure that distinguishes the Thai reimbursement process. The broader policy lessons, particularly the value of linking HTA to structured, deliberative priority setting, may offer relevant insights for other LMICs seeking to institutionalize HTA within universal health coverage frameworks.

## Data Availability

The data analyzed in this study is subject to the following licenses/restrictions: the datasets analyzed in this study are subject to institutional restrictions by the National List of Essential Medicines (NLEM) subcommittee. The data contain sensitive information related to pharmaceutical price negotiations and internal policy deliberations. Consequently, the data are not publicly available and can only be accessed by authorized personnel within the research and policy framework of the providing institutions. Requests to access these datasets should be directed to Rungpetch C. Sakulbumrungsil, rungpetch.c@pharm.chula.ac.th.

## References

[1] Organization for Economic Co-operation and Development. Pharmaceutical Innovation and Access to Medicines. Paris: OECD Publishing. (2019). Available online at: https://www.oecd.org/en/publications/pharmaceutical-innovation-and-access-to-medicines_9789264307391-en.html (Accessed February 23, 2026).

[2] World Health Organization. Health Intervention and Technology Assessment in Support of Universal Health Coverage. WHA67.23. Geneva: WHO. (2014). Available online at: https://apps.who.int/gb/ebwha/pdf_files/WHA67/A67_R23-en.pdf (Accessed February 23, 2026).

[3] O'Rourke B Oortwijn W Schuller T International Joint Task Group. The new definition of health technology assessment: a milestone in international collaboration. Int J Technol Assess Health Care. (2020) 36:187–90. doi: 10.1017/S0266462320000215 32398176

[4] Chalkidou K Glassman A Marten R Vega J Teerawattananon Y Tritasavit N . Priority-setting for achieving universal health coverage. Bull World Health Organ. (2016) 94:462–7. doi: 10.2471/BLT.15.155721 27274598 PMC4890204

[5] Glassman A Giedion U Smith PC. What's in, what's out: designing benefits for universal health coverage. Washington (DC): Center for Global Development. (2017). doi: 10.7490/f1000research.1114963.1

[6] Teerawattananon Y Tantivess S Yothasamut J Kingkaew P Chaisiri K. Historical development of health technology assessment in Thailand. Int J Technol Assess Health Care. (2009) 25:241–52. doi: 10.1017/S0266462309090709 19527543

[7] Tantivess S Teerawattananon Y Mills A. Strengthening cost-effectiveness analysis in Thailand through the establishment of the health intervention and technology assessment program. Pharmacoeconomics. (2009) 27:931–45. doi: 10.2165/11314710-000000000-00000 19888793

[8] Panwar V. Thailand's healthcare system and the role of HTA: a model of UHC [Internet]. P4H Network. (2024). Available online at: https://p4h.world/en/news/thailands-healthcare-system-and-the-role-of-hta-a-model-of-uhc/ (Accessed February 21, 2026).

[9] Paek SC Meemon N Wan TTH. Thailand's universal coverage scheme and its impact on health-seeking behavior. Springerplus. (2016) 5:1852. doi: 10.1186/s40064-016-3665-4 27933235 PMC5104696

[10] Teerawattananon Y Tritasavit N Suchonwanich N Kingkaew P. The use of economic evaluation for guiding the pharmaceutical reimbursement list in Thailand. Z Evid Fortbild Qual Gesundhwes. (2014) 108:397–404. doi: 10.1016/j.zefq.2014.06.017 25444298

[11] Glassman A Chalkidou K Giedion U Teerawattananon Y Tunis S Bump JB . Priority-setting institutions in health: recommendations from a center for global development working group. Glob Heart. (2012) 7:13–34. doi: 10.1016/j.gheart.2012.01.007 25691165

[12] Leelahavarong P Doungthipsirikul S Kumluang S Teerawattananon Y Inthadej P Chotivanechkoon K . Health technology assessment in Thailand: institutionalization and contribution to healthcare decision making: review of literature. Int J Technol Assess Health Care. (2019) 35:467–73. doi: 10.1017/S0266462319000321 31190670

[13] Mohara A Youngkong S Velasco RP Werayingyong P Teerawattananon Y Kumluang S . Using health technology assessment for informing coverage decisions in Thailand. J Comp Eff Res. (2012) 1:137–46. doi: 10.2217/cer.12.10 24237374

[14] Tanvejsilp P Werayingyong P Youngkong S Leelahavarong P Teerawattananon Y Tantivess S . Revisiting roles of health technology assessment on drug policy in universal health coverage in Thailand: where are we? and what is next? Value Health Reg Issues. (2019) 18:36–41. doi: 10.1016/j.vhri.2018.11.004 30641410

[15] Buxton M Hanney S. How can payback from health services research be assessed? J Health Serv Res Policy. (1996) 1:35–43. doi: 10.1177/135581969600100107 10180843

[16] Wooding S Hanney S Buxton M Grant J. Payback arising from research funding: evaluation of the arthritis research campaign. Rheumatology. (2005) 44:1145–56. doi: 10.1093/rheumatology/keh708 16049052

[17] Klautzer K Hanney S Pollitt A Grant J Buxton MJ. Assessing the “payback” of health research: a case study of the National institute for health research (NIHR) Health Technology Assessment (HTA) Programme. Santa Monica, CA: RAND Corporation. (2011). Report No.: RR-120-DH.

[18] Wooding S Hanney SR Pollitt A Grant J Buxton MJ Project Retrosight Team. Understanding factors associated with the translation of cardiovascular research: a multinational case study approach. Implement Sci. (2014) 9:47. doi: 10.1186/1748-5908-9-47 24755187 PMC4003508

[19] Stinnett AA Mullahy J. Net health benefits: a new framework for the analysis of uncertainty in cost-effectiveness analysis. Med Decis Making. (1998) 18:S68–80. doi: 10.1177/0272989X98018002S09 9566468

[20] Kim DD Basu A. New metrics for economic evaluation in the presence of heterogeneity: focusing on evaluating policy alternatives rather than treatment alternatives. Med Decis Making. (2017) 37:930–41. doi: 10.1177/0272989X17702379 28441507

[21] Kaur G Prinja S Lakshmi PVM Downey L Sharma D Teerawattananon Y. Criteria used for priority-setting for public health resource allocation in low- and middle-income countries: a systematic review. Int J Technol Assess Health Care. (2019) 35:474–83. doi: 10.1017/S0266462319000473 31307561

[22] Thokala P Devlin N Marsh K Baltussen R Boysen M Kalo Z . Multiple criteria decision analysis for health care decision making-an introduction: report 1 of the ISPOR MCDA emerging good practices task force. Value Health. (2016) 19:1–13. doi: 10.1016/j.jval.2015.12.003 26797229

[23] Butani D Faradiba D Dabak SV Isaranuwatchai W Huang-Ku E Pachanee K . Expanding access to high-cost medicines under the universal health coverage scheme in Thailand: review of current practices and recommendations. J Pharm Policy Pract. (2023) 16:138. doi: 10.1186/s40545-023-00643-z 37936171 PMC10631213

[24] Teerawattananon Y Dabak SV Culyer A Mills A Kingkaew P Isaranuwatchai W. Fifteen lessons from fifteen years of the health intervention and technology assessment program in Thailand. Health Syst Reform. (2023) 9:2330974. doi: 10.1080/23288604.2024.2330974 38715185

